# Does Context Count? The Association Between Quality of Care and Job Characteristics in Residential Aged Care and Hospital Settings: A Systematic Review and Meta-Analysis

**DOI:** 10.1093/geront/gnac039

**Published:** 2022-03-22

**Authors:** Batoul Hodroj, Kïrsten A Way, Theresa L Scott, April L Wright, Asmita Manchha

**Affiliations:** School of Psychology, University of Queensland, St Lucia, Queensland, Australia; Centre for Business and Organisational Psychology, School of Psychology, University of Queensland, St Lucia, Queensland, Australia; School of Psychology, University of Queensland, St Lucia, Queensland, Australia; Warwick Business School, University of Warwick, Coventry, UK; School of Psychology, University of Queensland, St Lucia, Queensland, Australia

**Keywords:** Burnout, Care quality, Counterproductive work behavior, Job demands, Job resources

## Abstract

**Background and Objectives:**

Within residential aged care settings, reduced quality of care (QoC), abuse, and neglect have been global phenomena which require urgent intervention. As the reported rate of these problems is much higher in aged care compared to hospital settings, we investigated whether differing job design characteristics between the 2 settings might explain the difference.

**Research Design and Methods:**

We used a meta-analysis to compare differences in the relationships between high job demands, low job resources, and job strain with QoC and counterproductive work behaviors (CWBs) across aged care and hospital settings.

**Results:**

Data were extracted from 42 studies (*n* = 55 effects). QoC was negatively correlated with high job demands ($\bar{\rho }$ = −0.22, 95% confidence interval [CI]: −0.29, −0.15, *k* = 7), low job resources ($\bar{\rho }$ = −0.40, 95% CI: −0.47, −0.32, *k* = 15), and job strain ($\bar{\rho }$ = −0.32, 95% CI: −0.38, −0.25, *k* = 22), CWBs had a positive relationship with job demands ($\bar{\rho }$ = 0.35, 95% CI: 0.10, 0.59, *k* = 3) and job strain ($\bar{\rho }$ = 0.34, 95% CI: 0.13, 0.56, *k* = 6). The association between poor QoC and low job resources was stronger in aged care (*r* = −0.46, 95% CI: −0.55, −0.36, *k* = 8) than in hospital settings (*r* = −0.30, 95% CI: −0.41, −0.18, *k* = 7).

**Discussion and Implications:**

Our findings suggest that relationships between low job resources and poor QoC are exacerbated in residential aged care contexts. To improve care outcomes, stakeholders should improve job resources such as skill discretion, supervisory supports, and increased training and staffing levels in residential aged care.

There is a growing awareness of abuse, neglect, and reduced quality of care (QoC) within residential aged care facilities. Prevalence rates of abuse targeted at older adults range from 6% to 82% internationally (Commonwealth of Australia, 2020) requiring urgent attention. Considering the global experience of abuse and neglect in residential aged care, this phenomenon is likely to be attributed to contextual factors in the industry. However, attempts to address the problem have focused on individual-level interventions that neglect factors at the organizational and field levels which may be enabling these negative outcomes (Montgomery et al., 2019). Therefore, to understand whether contextual factors unique to residential aged care are exacerbating incidents of abuse, neglect, and poor-quality care, comparing the relationship between these factors and outcomes in residential aged care with a similar care setting, such as hospitals, could assist.

Some of these contextual differences across care settings include key job characteristics and their associated social contexts. Job strain, for example, is particularly high in residential aged care compared to other industries (Dyrbye et al., 2017). Residential aged care facilities also have a reputation for being environments where staff are overworked and undervalued (Manchha et al., 2021; Nardi & Gyurko, 2013). Staff devaluation is especially concerning as a global increase in the aging population means that society’s reliance on residential aged care facilities will increase, with people aged 65 years or older doubling by 2050 to 26.1% (OECD, 2021). Scholars have emphasized the imperative for more research into the contextual factors that may influence employee behaviors and outcomes in residential aged care (Vogus et al., 2020).

This paper seeks to contribute to this agenda. We explore whether industry-level factors unique to residential aged care are exacerbating the impacts of job demands/resources and job strain on QoC, and on the onset of counterproductive work behaviors (CWBs; such as abuse and neglect). Using a systematic review and meta-analysis, this paper aims to: (a) aggregate through pooled effect sizes, existing research on the effects of job demands, job resources, and job strain on QoC, and CWBs in caregiving settings, and (b) investigate how the relationships between these antecedents and outcomes are moderated by the organizational settings of hospital and residential aged care.

This paper provides a foundational attempt to synthesize studies to explore sector-level differences in QoC and CWBs and seeks to explain potential reasons for these differences. While aspects such as ageism and social and cultural norms are explored as societal-level factors that may be affecting abuse and neglect in residential aged care (Pillemer et al., 2016), we suggest that the practical medium through which these behaviors manifest are institutional logics operating in the field of aged care. In our literature review, we theorize that differences in setting may be explained by distinctive market and professional institutional logics associated with the residential aged care field that influence work design under the job demands–resources model (Demerouti et al., 2001; Kurtmollaiev et al., 2018). This understanding will be critical in informing the conversation about factors relevant stakeholders should focus on to improve QoC and prevent ongoing abuse and neglect in residential aged care.

## Theoretical Underpinnings of the Current Study

We propose, based on institutional logic theory, that the guiding rules and assumptions within residential aged care are influenced by strong market logics and weak professional logics. Institutional logic theory posits that organizational fields, such as residential aged care, are influenced by social assumptions and norms that create and influence the inter- and intrasystems of that field (Powell & DiMaggio, 1991) and affect organizational decision making. As such, factors unique to the organizational field of residential aged care may influence work designer decisions to create an environment where there are high job demands, low job resources, and higher incidences of job strain. These unique factors may be an increased market logic and weakened professional logic that may affect QoC and incidents of CWBs, such as abuse and neglect.

Within the context of residential aged care, it is reasonable to propose that a market logic would drive high job demands and low investment of resources. A market logic stipulates that organizations are influenced by a transactional approach where efficiency and profit drive the provision of services to individuals (Campbell & Pedersen, 2001). Profit-driven services are prominent given that residential aged care has high levels of privatization globally (King, 2007). Approximately 99% are privately owned in the United States (National Center for Health Statistics, 2016), 86% in the United Kingdom, 98% in Germany, 90% in Australia, and similar patterns in other countries underpinned by neoliberal ideologies (Dyer et al., 2019). Within aged care, the promotion of marketization has favored complicated systems that promise consumer choice over the fair and equitable delivery of quality services for all older adults (Brennan, 2012). Job quality, characterized by adequate pay, high training, skill requirements, and autonomy, is lower under marketized regimes, compared to inclusivist regimes where there are strong policies related to worker rights and employee conditions (Holman, 2013). Considering this, job characteristics in residential aged care may be affected more strongly by market logics compared to hospital environments where there is a smaller privately owned sector (approximately 15% in the United Kingdom, 17% in Germany [Commonwealth Fund, 2021], 49% in Australia [Australian Bureau of Statistics, 2018]).

Additionally, in residential aged care, the stigma associated with working with older adults in need of 24-hr care may be affected by marketization at a societal level. Where a neoliberal society values independence and individual contribution to economic goals, older adults in need of care may be perceived as an economic burden (Banks, 2018). The willingness to invest in care for residents in aged care may be weaker compared to patients who are treated in hospitals who have typically short-term stays with some form of recovery. Due to this, we argue that even in countries where the disparity in privatization of aged care versus hospitals is not as stark (e.g., in the United States where 99% of residential aged care vs 81.5% of hospitals are privately run; American Hospital Association, 2021), the effect of the market logic will still drive higher job demands and lower investment of resources in aged care.

In addition, a dominant market logic in residential aged care may weaken the professional logic for frontline workers (e.g., personal carers, nurses). A professional logic relates to the norms, social assumptions, and rules which increase personal reputation and status within, and of, the profession (Thornton & Ocasio, 2008). These logics may affect the two care settings via structural differences in skill sets and staff mixes (Phillips et al., 2006), as well as greater investment in training, a stronger multidisciplinary approach, and greater union and professional association representation and advocacy in hospital settings. This may exacerbate job demands and reduce the level of job resources available in residential aged care when compared to hospital settings.

Currently, there are limited studies that quantitatively measure the direct impact of institutional logics on negative outcomes in residential aged care. However, given the existing research related to the relationships between market and professional logics and worker outcomes such as training, pay, and staffing levels (Westermann-Behaylo, 2014), we argue that these macrocontextual factors may be creating differences in negative outcomes between residential aged care and comparable settings. This could be observed through the differential impact of job demands/resources on care quality, abuse, and neglect between residential aged care and hospitals.

### QoC in Residential Aged Care and Hospitals and Its Links With Job Demands, Job Resources, and Job Strain

We use Demerouti et al.’s (2001) JD-R to understand the relationships between job characteristics and the negative outcomes of poor-quality care, abuse, and neglect. JD-R posits that high job demands (such as workload) and low job resources (such as autonomy, social supports) can cause workers to feel overloaded. Within care settings, an increase in job demands and a lack of job resources limit the capacity for workers to engage in an individualized approach to care, reducing the QoC provided (Almost & Laschinger, 2002). The imbalance between demands and resources may have direct implications for the negative outcomes occurring within residential aged care and hospital settings.

*Job demands and resources* have been shown to affect a range of outcomes in care settings. *Job demands* in care settings can include the emotional demands of the work, role overload due to insufficient staffing levels, time pressures, role conflict, and work-related interpersonal conflict (Peters et al., 2012). The negative implications of job demands include lower levels of job satisfaction, turnover, and increased burnout (Scanlan & Still, 2019) and are negatively associated with QoC (Sarafis et al., 2016). *Job resources*, in contrast, are the social, physical, and psychological aspects of a role that improve role performance (Bakker et al., 2004). Some key job resources in residential aged care include supervisory and coworker support, flexibility with work schedules, dementia training, and meaningful sustained relationships with residents (Chenoweth et al., 2010). These resources increase patient-centered care (Almost & Laschinger, 2002). While the relationships between job demands/resources and care quality seem well established in the literature, the relative strength of job demands/resources on person-centered care quality is yet to be established through meta-analysis. We hypothesize that:

H1: Lower QoC in care settings will be predicted by higher job demands (1a), and lower job resources (1b).

Furthermore, distinguishing between job demands/resources and the psychological impact of this imbalance, job strain will be important to analyze. *Job strain* has been conceptualized as the negative psychological and physiological responses to work stressors (Demerouti et al., 2001). Job strain can also be operationalized using a range of job domain-specific psychological well-being measures such as burnout, job stress compassion fatigue, and feelings of hopelessness related to one’s job (Stamm, 2005). High job strain has also been shown to reduce the ability of health care workers to provide patient-centered care (Hunter et al., 2020). However, there have been mixed findings related to the impact of job strain on care quality. For example, Tawfik et al. (2019) found that burnout predicted poor-quality care in health care settings in almost half the studies identified in their meta-analysis but showed no effect or a small positive effect in the other half of studies identified, with the overall finding suggesting that burnout is frequently associated with poor-quality care. We will extend this research by including additional measures of job strain, such as burnout and job stress, on QoC, hypothesizing that:

H2: Lower QoC in care settings will be predicted by higher job strain.

### CWB and Its Links to Job Demands, Job Resources, and Job Strain

Incidents of abuse and neglect in residential aged care can be conceptualized as a CWB. CWBs are intentional acts carried out by employees that negatively affect the interests of the organization they work for (Sackett & DeVore, 2001). Within aged care, abuse toward residents can be conceptualized as an interpersonal deviant behavior and a subset of CWBs. Abuse has been linked to situational and environmental variables such as organizational culture and stressful work conditions (Spector et al., 2006). Similarly, production deviance or refusing to perform at the stated standards prescribed by the role is a form of passive sabotage due to worker anger or stress in response to organizational factors (Spector et al., 2006). Production deviance can manifest as withholding effort, absenteeism, or presenteeism within care settings (Carpenter & Berry, 2017). Failure to perform caretaking duties through production deviance can be considered a form of neglect (Pillemer et al., 2016). Among other things, the onset of CWBs can occur in reaction to a shortage of organizational resources, or increased job demands (Balducci et al., 2011). For this reason, it is expected that the high incidents of abuse and neglect reported in residential aged care settings could be attributed to high demands coupled with low resources. CWBs are also likely to occur in response to increased job strain (Zaghini et al., 2020). In our study we aim to meta-analyze effects of work design factors on CWBs (including production and interpersonal deviance) in care settings, hypothesizing that:

H3: Higher CWBs in care settings will be predicted by higher job demands (3a), and lower job resources (3b).

H4: Higher CWBs in care setting will be predicted by higher levels of job strain.

### Moderating Effect of Residential Aged Care Versus Hospital Settings

While it is useful to understand the impact of job demands, resources, and strain on negative work outcomes, we want to determine whether the relationship between these variables is stronger in residential aged care. Current reports indicate that poor QoC and CWBs such as abuse and neglect are more frequent in residential aged care settings (Andela et al., 2018a) compared to hospital settings but these findings have not been synthesized across multiple studies. This potential moderating effect is important to explore due to significant negative implications for the workforce. For example, when interviewed, nurses who work within aged care state that they find their work less worthy, even though the role description should be comparable to those of nurses working in hospital settings (Venturato et al., 2006). Nurses were also found to demonstrate a higher aversion to work in aged care compared to hospital settings (Eley et al., 2007) and experience a higher rate of burnout (McHugh et al., 2011). In line with our theorizing, we hypothesize that:

H5a: The negative relationship between caring behavior and high job demands–low resources will be stronger in residential aged care than in hospital care settings.

H5b: The negative relationship between caring behavior and job strain will be stronger in residential aged care than in hospital care settings.

H6a: The positive relationship between CWBs and high job demands–low resources will be stronger in residential aged care than in hospital care settings.

H6b: The positive relationship between CWBs and job strain will be stronger in residential aged care than in hospital care settings.

## Methodological Purpose of Using a Meta-Analysis

While we acknowledge that there are meta-analyses that have researched whether QoC is predicted by employee burnout (see Tawfik et al., 2019), our study continues this academic conversation and adds to the body of knowledge in several important ways. First, our meta-analysis has a broader scope, synthesizing studies that measured the relationships between care and a broader range of predictors (job strain, and job stress in addition to burnout as well as high job demands and low job resources). The inclusion of CWBs as an outcome variable is also a new and novel contribution with practical implications. Additionally, Tawfik’s et al.’s (2019) criteria for what is considered QoC relates to the completion of physical and administrative aspects of care such as medical error rates, infection control, accurate diagnosis, and adequate discharge practices. While these are crucial aspects to care, our meta-analysis focuses on the quality of person-centered care. A person-centered approach to care refers to a type of care that is tailored to the individual residents’ needs and uses communication and collaborative engagement with the client that values their autonomy and subjective experience (Edvardsson et al., 2010). The completion of tasks does not mean that residents or patients are receiving a quality person-centered experience, which is particularly necessary for long-term care in residential aged care facilities (Cleland, 2021). Last, and most importantly, our primary aim was to assess any moderating impact of different care settings. This has not been investigated in the academic literature to date.

## Method

We conducted a systematic review and random-effects meta-analysis to compare the pooled effect sizes of the relationships of high job demands, low job resources, and job strain with employee outcomes of QoC and CWBs across residential aged care and hospital settings. We registered our protocol with PROSPERO (CRD42020145951). We used PRISMA guidelines to ensure all appropriate information was included in the reporting of this paper (see Supplementary Materials).

### Search Strategy

We searched Web of Science, CINAHL, Scopus, ABI/Inform/ProQuest, and Medline in July 2019, September 2020, and again in August 2021. Search alerts were set up to monitor any new publications between search and time of publication. Additional papers were sourced through searching the reference lists of review articles which researched similar topics.

### Inclusion and Exclusion Criteria

We used the PICO (population, independent variable [predictor], comparison, outcome) framework to create our inclusion and exclusion criteria for the search and screening. A full list of search terms is provided in Table 1 and Supplementary Materials. The population of interest included workers in hospital and residential aged care settings (such as nurses and personal carers). The predictor variables included job demands, job resources, and employee job strain. The comparison of interest was between residential aged care and hospital care. Finally, the outcomes were QoC and CWBs. We excluded papers that were qualitative or did not include usable effect sizes. We contacted authors for missing data and included the useable replies (those that included a correlation coefficient) in our data analysis. We used Google to translate non-English papers to extract correlations, excluding those that could not be easily interpreted (including a total of three non-English papers). There were no restrictions on year of publication.

**Table 1.** Summary of Included Studies

Study	*n*	Setting	Predictor	Predictor description	Outcome	*r*	α (IV)^a^	α (DV)^a^
Abdelhadi and Drach-Zahavy (2012)	158	Aged care	Low job resources	Service climate	QoC	−0.26	0.93	0.87
Abekah-Nkrumah and Nkrumah (2021)	179	Hospital	Low job resources	Supervisor support, coworker support	QoC	−0.41	0.89	0.82
Alexiou et al. (2021)	62	Aged care	Job strain	Strain in Nursing Assessment Scale (SNCS)	QoC	−0.31	0.80	0.80
Alhalal et al. (2020)	255	Hospital	Job strain	Burnout—Professional Quality of Life Scale (ProQol)	QoC	−0.22	0.94	0.82
			Low job resources	Structural empowerment using the Conditions of Work Effectiveness Questionnaire-II (CWEQ-II)	QoC	−0.37	0.90	0.84
Andela et al. (2018b)	481	Aged care	Job strain	Maslach Burnout Inventory (MBI)—Emotional Exhaustion	CWBs	0.41	0.87	0.81
			Job demands	Workload; emotional demands	CWBs	0.29	0.78	0.81
			Low job resources	Quality of colleague relationships and supervisor relationships	CWBs	0.34	0.84	0.81
Bachnick et al. (2018)	1,810	Hospital	Low job resources	Nurse manager ability, leadership, and support of care workers and staffing and resource adequacy	QoC	−0.30	n/a	n/a
Backman et al. (2016)	3,661	Aged care	Low job resources	Psychosocial climate in terms of safety, everydayness (i.e., environment having a near and everyday character), and community (i.e., social contacts available to the residents)	QoC	−0.54	n/a	0.77
Basar and Basim (2016)	456	Hospital	Job strain	Malach-Pines (2005)—Burnout	CWBs	0.64	0.81	n/a
Bégat et al. (2004)	138	Hospital	Job strain	Work Environment Questionnaire (WEQ)—Job stress and anxiety	QoC	0.20	0.91	0.73
			Job demands	Work Environment Questionnaire (WEQ)— Work demands and lack of time	QoC	0.03	0.91	n/a
Burtson and Stichler (2010)	126	Hospital	Job strain	Average effect of Professional Quality of Life Scale (ProQol) and SIG Scale total	QoC	−0.23	0.81	0.92
Caspar and O’Rourke (2008)	568	Aged care	Low job resources	Information, support, resources, and opportunity	QoC	−0.32	n/a	n/a
Caspar et al. (2017)	131	Aged care	Low job resources	Supervisor support	QoC	−0.50	0.96	0.90
Chana et al. (2015)	102	Hospital	Job strain	Maslach Burnout Inventory (MBI)—Emotional Exhaustion	QoC	−0.31	0.9	0.96
Chao et al. (2016)	98	Hospital	Job strain	Maslach Burnout Inventory (MBI)—Occupational Burnout	QoC	0.12	n/a	n/a
El-Hneiti et al. (2019)	500	Hospital	Job demands	Violence from patients	QoC	−0.17	n/a	n/a
Fagbenro (2019)	300	Hospital	Job demands	Role ambiguity	CWBs	0.48	0.86	0.86
Fopma-Loy (1991)	107	Aged care	Job strain	Formal Caregiver Stress Instrument	QoC	−0.18	n/a	n/a
Gountas et al. (2014)	159	Hospital	Job strain	Maslach Burnout Inventory (MBI)—Emotional Exhaustion	QoC	−0.20	0.83	0.91
Harris and Artis (2005)	113	Hospital	Job strain	Patient Burnout (PBO)	QoC	−0.20	0.7	0.70
Jamal (1984)	440	Hospital	Job demands	Role ambiguity, role overload, role conflict, resource inadequacy	QoC	−0.33	n/a	0.91
Jarrar et al. (2019)	652	Hospital	Job demands	Excessive work hours	QoC	−0.15	n/a	n/a
Kaur et al. (2013)	448	Hospital	Job strain	Maslach Burnout Inventory (MBI)—Burnout composite	QoC	−0.22	n/a	n/a
Kazemi and Elfstrand Corlin (2021)	1,342	Aged care	Low job resources	Supportive leadership practices	QoC	−0.36	0.92	0.84
Kim and Park (2019)	171	Aged care	Job strain	Stress for elderly care captured through task stress, patient stress, and caregiver stress	QoC	−0.09	0.65	0.86
			Low job resources	Nurse participation in hospital affairs, nursing foundations for QoC, nurse manager ability, leadership, and support of nurses, staffing, and resource adequacy, collegial nurse–physician relations	QoC	−0.63	0.84	0.86
Lee and Pak (2016)	422	Hospital	Low job resources	Social support from colleagues	QoC	−0.14	n/a	n/a
Lee (2004)	770	Hospital	Job demands	Job demands—uncertainty concerning treatment, conflict with physicians, conflict with supervisors or other nurses, lack of support, workload, inadequate preparation and witnessing patient’s suffering death, and dying	QoC	−0.15	0.77	n/a
Liu and Aungsuroch (2017)	510	Hospital	Job strain	Maslach Burnout Inventory (MBI)—Burnout composite	QoC	−0.39	0.93	n/a
			Low job resources	Resource adequacy, nurse–physician relations, nurse managers ability, leadership, and support for nurses and nursing foundations for QoC	QoC	−0.46	0.91	n/a
McLinton et al. (2019)	420	Hospital	Job demands	Emotional demands	CWBs	0.30	0.82	n/a
					QoC	−0.40	0.82	n/a
	419	Hospital	Low job resources	Skill discretion	CWBs	−0.02	0.68	n/a
					QoC	−0.06	0.68	n/a
	435	Hospital	Job strain	Maslach Burnout Inventory (MBI)—Burnout composite	CWBs	0.14	0.89	n/a
McNeil et al. (2019)	194	Aged care	Job strain	Maslach Burnout Inventory (MBI)—Burnout composite	QoC	−0.27	0.83	0.92
			Low job resources	Coworker support, team cohesion	QoC	−0.27	0.93	0.92
Motowidlo et al. (1986)	171	Hospital	Job strain	Bespoke measurement	QoC	−0.22	0.83	0.81
Neuberg et al. (2017)	171	Aged care	Job strain	Maslach Burnout Inventory (MBI)—Emotional Exhaustion	QoC	−0.16	n/a	n/a
					CWBs	0.05	n/a	n/a
Panunto et al. (2013)	129	Hospital	Job strain	Maslach Burnout Inventory (MBI)—Emotional Exhaustion	QoC	−0.44	0.9	n/a
Rathert et al. (2020)	631	Hospital	Job strain	Maslach Burnout Inventory (MBI)—Emotional Exhaustion	QoC	−0.27	0.91	0.87
Sarafis et al. (2016)	246	Hospital	Job strain	Expanded Nursing Stress Scale (ENSS)	QoC	−0.30	0.96	n/a
Sarwar et al. (2021)	295	Hospital	Job strain	SIG Scale total	CWBs	0.25	n/a	n/a
Shafique et al. (2020)	417	Hospital	Job strain	Maslach Burnout Inventory (MBI)	CWBs	0.37	n/a	n/a
Skoldunger et al. (2020)	3,605	Aged care	Job strain	Job strain measured on the Demand-Control- Support Questionnaire (DCSQ)	QoC	−0.42	n/a	n/a
Sullivan et al. (2019)	723	Aged care	Low job resources	Job resources such as collaborative influence (i.e., one’s influence over other care providers’ decisions, supportive organizational context, and supervisory support)	QoC	−0.37	0.91	0.67
Van Bogaert et al. (2009)	401	Hospital	Job strain	Maslach Burnout Inventory (MBI)—Emotional Exhaustion	QoC	−0.35	0.88	n/a
			Low job resources	Hospital management and organizational support Nurse–physician relationship Nurse management	QoC	−0.37	0.75	n/a
Van Bogaert et al. (2013)	1,201	Hospital	Job strain	Maslach Burnout Inventory (MBI)—Emotional Exhaustion	QoC	−0.29	n/a	0.79
Van Diepen et al. (2021)	94	Hospital	Job strain	Demand-Control-Support Questionnaire	QoC	0.43	0.69	0.80
Zuniga et al. (2015)	4,294	Aged care	Job demands	Conflict and lack of recognition, workload, and lack of preparation	QoC	−0.23	n/a	0.69

*Notes*: CWB = counterproductive work behaviors; QoC = quality of care. The IV description lists the scales used for job strain and describes the specific job demand and job resource variables measured in each study. Scales are not listed for QoC and CWBs as we are not interested in the various types of person-centered care or CWBs for this study.

^a^Not all studies reported the reliability of their measures (Cronbach’s α), and this is reflected as n/a.

After extracting articles from each database according to the search criteria, we removed duplicates and imported the articles into screening software. Two screeners used a three-stage screening process which involved first screening title and abstract against the PICO inclusion and exclusion criteria. A full-text screen where the whole paper was read and evaluated against the inclusion and exclusion criteria was then conducted. Finally, studies that featured unusable data were excluded. All studies that provided a Pearson’s or Spearman’s correlation were included. Interrater agreement for full-text screening was calculated using Cohen’s kappa coefficient. Agreement between the two screeners for the July 2019 screening was Cohen’s *k* = 0.95, 97.56% agreement, and Cohen’s kappa was 0.62; 81.61% agreement for the September 2020 screening. The August 2021 search revealed four additional studies. Disagreements between screeners were discussed and resolved in a consultative fashion.

**Figure 1.**
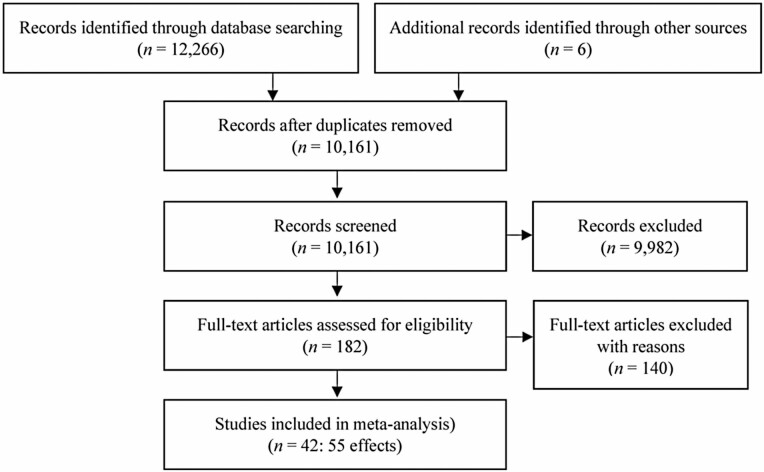
Study selection based on the inclusion and exclusion criteria.

As seen in Figure 1, we initially identified 12,266 papers and removed 1,462 duplicates. After title and abstract screening, we excluded 9,982 papers that did not meet the inclusion criteria and conducted a full-text screening of 182 papers. The total number of papers that met inclusion criteria for data extraction was 42 (*n* = 55 effects; see Table 1). Studies were assessed for their quality with 34 found to be of high quality, seven of moderate quality, and one of low quality (see Table 2; Supplementary Materials; Adams et al., 2018).

**Table 2.** Meta-Analytic Estimates of Correlations Among Job Demands, Job Resources, and Job Strain With Quality of Care and Counterproductive Work Behaviors

Predictor and outcome variables	*k*	*n*	$\bar{\rho }$ , *SD*	95% CIs	*I* ^2^
Quality of care outcome					
Job demands	7	7,302	−0.22, 0.08	[−0.29, −0.15]	86.71
Low job resources	15	10,944	−0.40, 0.14	[−0.47, −0.32]	95.18
Job strain	22	9,132	−0.32, 0.15	[−0.38, −0.25]	90.72
Counterproductive work behaviors outcome					
Job demands	3	1,201	0.35, 0.10	[0.10, 0.59]	94.73
Job strain	6	2,255	0.34, 0.20	[0.13, 0.56]	95.03

*Notes*: CI = confidence interval; *SD* = standard deviation. *I*^2^ > 75 indicates high heterogeneity. Job resources are not included in this table as a predictor of counterproductive work behaviors as there were only two studies that reported effect sizes for this relationship.

### Data Extraction and Coding Procedure

We extracted variables that met the inclusion criteria for job demands, or job resources, according to definitions described by Bakker and Demerouti (2007), or as determined by the coding template and verified by content experts. Where multiple job demands (or job resources) were reported in one paper, we aggregated the reported correlations to create one job demands (or job resources) variable. Job resources were coded in the direction of lack of job resources to stay consistent with our hypothesizing. This meant that positive correlations related to job resources were reversed to negative correlations to represent lack of job resources. Studies that measured QoC through scales measuring “poor-quality care” were also reverse-coded by changing the associated sign (direction) of the correlation.

The variable of job strain included data from studies which reported measures of job strain, job stress, or burnout (see Table 1). Where the emotional exhaustion subscale of burnout was reported, this was extracted as the measure of job strain. This was due to emotional exhaustion being recognized as the core construct within burnout (Maslach et al., 2008; West et al., 2012). Where studies did not report subscale scores for burnout, we extracted the full burnout measure as the indicator of job strain.

Settings were considered hospital care if they were described as containing the main components of acute hospital settings such as intensive care units, emergency departments, medical wards, surgical wards, and acute clinical units. Settings were considered residential aged care facilities if they were described as such, or with related terms such as long-term care, nursing home facility, rest home, or care home, where the main objective of the institution was to care for older people for an extended time period in a residential setting. All measures of person-centered care, individualized care, QoC, or caring behaviors were coded as the outcome variable, QoC. Measures of physical care or patient safety, such as medical errors, were not included as these were not consistent with our conceptualization of person-centered care.

CWBs were coded using categorizations outlined in Spector et al. (2006) model (i.e., production and interpersonal deviance). This means abuse and neglect, as well as presenteeism and absenteeism, were included in our conceptualization of CWBs. Where single studies reported multiple types of CWBs, we aggregated these into one effect size.

### Analytic Procedure

We conducted a random-effects meta-analysis for main and moderation effects. For main effects we aggregated effect size estimates using two methods. The first method was based on Schmidt and Hunter (2015) where correlations from all studies are aggregated to assess the association between the outcome variables (i.e., QoC and CWB) and the predictor variables (i.e., job demands/resources and job strain). Schmidt and Hunter (2015) utilize meta-analytic techniques that are more appropriate for research based on psychometric data, as population and measurement biases and variability are required to be accounted for in psychological research. Through the process of meta-analysis, this approach uses the study sample size as a weight of effect size estimates in combination with accounting for variability in each study that is due to measurement and sampling error. The true effect size estimate for each relationship is weighted based on the formula $W_{i}=N_{i}*{A_{i}}^{2},$ where $A_{i}$ is the attenuation factor (or the sample-specific bias due to measurement error) and this was the formula used in the package in R, used in our analysis. Where studies reported multiple effect sizes for the one variable (e.g., multiple job resources and their relationship with caring behavior), the correlation coefficients were averaged to produce a single effect size for that study. Due to potential issues with dependency of effect sizes and multiplicity of effects, and in line with best practice recommendations (Rudolph et al., 2020), we also utilized a second method (robust variance estimates) to compute the correlation effect size based on Tanner-Smith and Tipton (2014), which accounts for this problem.

Regular assumption checks for heterogeneity in multiple regression were conducted by checking the *I*^2^ statistic (Huedo-Medina et al., 2006). An *I*^2^ statistic of less than 75% suggests that the assumption of heterogeneity is not violated. We also checked for publication bias by visually assessing funnel plots. A roughly symmetrical upside-down funnel with effect sizes scattering to the left and right of the main effect size indicates that there is no publication bias (Harrer et al., 2021).

We conducted a meta-regression to assess for moderation effects. We did this by using setting as a moderator variable in the regression model and then conducting a subgroup analysis for significant differences. That is, we observed whether there was also a significant confidence interval (CI) overlap of the effect sizes between hospital setting and aged care setting.

These analyses were conducted in R using the statistics packages *psychmeta, metafor*, and *robumeta*. The data set can be found in Table 1.

## Results

### Assumptions and Biases

Assumption checks suggest that there is a considerable level of heterogeneity shown by an *I*^2^ of greater than 75% (see Table 2). This means that variability in the effect sizes for the included studies may be due to effects other than random error (Borenstein et al., 2021). To try and account for this potential moderator we conducted a moderation analysis (using meta-regression) investigating whether differences in the way job strain was measured accounted for this variability. We conducted this analysis using the sample with the highest number of studies (QoC and job strain, *k* = 22). This moderation was not significant, and results can be found in Supplementary Materials.

We tested for publication bias by visually assessing the effect sizes on funnel plots. Visual analysis (see Figure 1; Supplementary Materials) shows a varied and symmetrical distribution of effect sizes on either side of the funnel, indicating an absence of publication bias. However, many of the studies also fall outside of the vertical lines on either side. This is expected due to the high level of heterogeneity and low number of effect sizes (IntHout et al., 2015).

### Main Effects

Supporting H1a and H1b we found lower QoC was predicted by high job demands and low job resources. We found support for H2 that lower QoC was predicted by higher levels of job strain across the full sample. We found support for H3a with CWBs being predicted by high job demands. Unfortunately, we could not conduct an analysis between CWBs and job resources, as we only had two studies which fell below the acceptable number for meta-analysis (*k* = 3; Rudolph et al., 2020). We found support in line with our hypothesis (H4) that higher incidences of CWBs were predicted by higher levels of job strain in the full sample. All effect size estimates for our main effects can be found in Table 2. All supported effects noted above were also supported by the robust variance estimate which indicates a significant effect (Table 3). Forest plots for all effects can be found in Supplementary Materials.

**Table 3.** Meta-Analytic Robust Variance Estimates of Correlations Among Job Demands, Job Resources, and Job Strain With Quality of Care and Counterproductive Work Behaviors

Predictor and outcome variables	*r*	*SE*	*t*	95% CIs	*p* Value
Quality of care outcome					
Job demands	−0.22	0.05	−4.34	[−0.34, −0.09]	.000
Low job resources	−0.39	0.04	8.66	[−0.48, −0.29]	.005
Job strain	−0.22	0.05	−5.08	[−0.31, −0.13]	.000
Counterproductive work behaviors outcome					
Job demands	−0.37	0.07	5.03	[0.05, 0.69]	.038
Job strain	0.34	0.10	3.35	[0.08, 0.61]	.020

*Notes*: CI = confidence interval; *SE* = standard error.

### Moderation Effects

Our moderation analysis using meta-regression found that setting was a significant moderator of the association between QoC and job resources, *F*(1, 2) = 3.92, *p* = .047, partially supporting H5a. Using subgroup analysis, we determined that the association between QoC and low job resources is stronger in residential aged care (*r* = −0.46, *SD* = 0.11, 95% CI [−0.55, −0.36], *k* = 8) than in hospital care settings (*r* = −0.30, *SD* = 0.13, 95% CI [−0.41, −0.18], *k* = 7). Due to there being only one study in aged care that measured the association between QoC and job demands, we were unable to run a moderation analysis for this aspect of H5a. Setting was a nonsignificant moderator for the relationship between QoC and job strain, *F*(1, 2) = 0.54, *p* = .46.

Further analysis of possible moderation effects of aged care and hospital care for the outcome variable of CWBs was not able to be conducted as studies fell below the acceptable number (*k* = 3; Rudolph et al., 2020).

## Discussion

Our meta-analysis aimed to test the main and moderating effects between the response variables of QoC and CWBs and predictor variables of job demands, job resources, and job strain across residential aged care and hospital settings. While there were some limitations in the number of analyses that could be conducted due to small sample size, our tested hypotheses were largely supported.

### Impacts of Job Demands, Job Resources, and Job Strain on QoC

In support of our hypotheses, poor QoC was predicted by high job demands, low job resources, and job strain, and CWBs were predicted by high job demands and job strain. This supports our theorizing in line with the JD-R model of work design where high levels of job demands, and low job resources negatively affect job performance (Bakker, 2004), which in the case of carers, relates to their performance in providing care. Our results support that this also occurs for job strain and may limit worker capacity to focus on their duties, particularly if their role requires emotional resources to provide patient-centered care, such as empathy, understanding, active listening, and emotional regulation (Kinman & Leggetter, 2016).

#### Strength of relationships in predicting outcomes

Interestingly, we found that poor QoC was predicted more strongly by low job resources than by high job demands (−0.40 vs −0.22). This suggests that while job demands reduce the capacity of workers to provide quality care, a lack of access to job resources (such as decision-making power, training and development opportunities, and work-related social support) has a greater impact on caring behavior (Gao et al., 2014). In support of this, Lesener et al. (2019) found that low job resources predicted low work engagement over time more significantly compared to high job demands. These findings endorse the importance of investment in job resources, particularly in highly demanding care roles.

### Impacts of Job Demands, and Job Strain on CWBs

We expected that job demands, low job resources, and job strain would have a positive relationship with CWBs. Our hypothesis was supported for job demands and job strain, but we were unable to test effects for job resources. This suggests that workers engage in behaviors that are in opposition to organizational expectations, in response to the demands and level of strain placed on them by the organization. Our findings are supported by Balducci et al.’s (2011) research, where job demands were found to increase abuse/hostility CWBs. Job demands were also found to be situational triggers that activate CWBs in certain individuals (O’Brien et al., 2021). This supports our theoretical focus on work design as a point of intervention for abuse and neglect in care settings. That is, if organizational-level factors such as job demands are significantly impactful in facilitating CWBs, then a focus of policy and research into the contextual factors that increase CWBs in care is imperative. Further research is required to be able to assess the role of low job resources on CWBs in health care settings at a meta-analytic level.

### Moderating Effect of Residential Aged Care Versus Hospital Settings

Of main interest to the research agenda for this paper, we found support for our hypothesis that the strength of the relationship between job resources and QoC was stronger in residential aged care than in hospital settings. This finding is in line with our theorizing that institutional logics affect the job resources available to residential aged care workers. Within residential aged care, it is likely that there is low decision latitude, low job complexity, and low autonomy, and also less access to tangible job resources (such as adequate staffing ratios, training, professional support, and worker advocacy) to support frontline staff (Gao et al., 2014). We propose that because market logics may serve to devalue aged care workers, that there is low justification for organizations to invest in wages, skill development, and minimum staffing ratios despite the ongoing abuse, neglect, poor well-being, and turnover in the industry (Gao et al., 2014). Within marketized neoliberal structures, resource allocation may be prioritized toward those who operate the core business model and financial operations (Mumby, 2019). These logics may be less prevalent in hospital settings.

Interestingly, we did not find support for our hypothesis that the relationship between job strain and caring behavior will be stronger in residential aged care compared to hospital care. This suggests that the relationship between QoC and level of job strain in different contexts may be complex and have other unaccounted-for interactions. This was noticed in the data where in a few studies, person-centered care had a positive relationship with job strain. For example, in Van Diepen (2021), person-centered care was associated with higher levels of job strain, but lower moral distress and intention to leave. Here, the behavior of person-centered care was perceived as resource-intensive (as greater time and skill is required). While this resulted in higher levels of strain, it mitigated other stressors related to care, such as moral discomfort at work from providing lower standards of care. In addition to such factors, Van Diepen (2021) conducted their study cross-sectionally during an intervention change period with high supports. As such the intensiveness of learning a new skill could have contributed to the higher feelings of strain, but generated positive outcomes through job complexity (Chung-Yan, 2010). Future studies should explore this longitudinally and discuss the differences between short-term experiences of job strain, and job strain experienced over an extended period of time.

### Practical Implications for Residential Aged Care

Our findings indicate that organizational setting (residential aged care) exacerbated the negative implications of low job resources on QoC. This suggests that the impact of broader market and professional logics on work design decisions in aged care settings is important to explore. Specifically, policy and government decisions regarding privatization and resource models point to possible reasons for reduced resourcing in the field of residential aged care (Casey et al., 2011). Our results support that governments and providers need to consider the impact of a marketized mentality on the value attribution and resource allocation provided to workers in residential aged care services.

Practically, this speaks to two levels of potential change for organizations and policy makers seeking to improve the QoC outcomes in residential aged care. The first can be targeted at the managerial level where individual agents or actors are able to alter work design for frontline staff in aged care that focuses on resource availability and job enrichment. The impact of institutional logics on business models can vary from sector to sector, and key institutional actors with power and decision-making capabilities could consider job redesign to enrich work for those on the front line (Gog et al., 2018) and reduce engagement in CWBs. Specific and practical intervention points where managers could improve conditions include job resources such as skill discretion, supervisory supports, and increased training and staffing levels and reduce job demands such as high workloads, excessive work hours, and emotional demands.

The second is targeted at the system level where operational and government stakeholders could consider which dominant logics guide their operations and take a purposeful approach to alter these logics. For example, Reay and Hinings (2005) discuss institutional change in health care in Alberta from a structural system based on an illness model to one based on a “wellness” model. This change entailed altering logics at the state, administrative, and professional education levels through a staged approach over a decade. Successful change hinged on identifying key actors within the field—including representatives from the government, nurses, and physicians—to advocate and implement purposeful changes in policies, attitudes, and administrative structures geared toward a focus on client well-being. While extensive and laborious, our results suggest a similar systemic shift is necessary in residential aged care with a focus on greater job resources.

### Limitations

This study has several limitations. First, we were unable to conduct interpretable moderation analysis for the impact of setting on the relationship between CWBs and job demands, job resources, and job strain due to the small number of effect sizes. Second, while there seems to be a considerable level of research in hospital settings, fewer studies have been conducted in residential aged care. Third, a key limitation is the unaccounted-for heterogeneity in the papers in our sample that we were unable to explain. While it is important for psychological research to ensure that comparisons between groups control for extraneous variables (Linden & Hönekopp, 2021), high heterogeneity is not unusual in meta-analyses related to occupational stress and in organizational settings, particularly if the moderating variables are yet to be measured and those settings are conducted in nonexperimental environments (Asif et al., 2018). The small *k* (number of studies) also means that high heterogeneity is more likely. While we have attempted to account for multiplicity, the increased likelihood of a Type I error was a limitation due to the analysis of different associations through six separate meta-analysis, with some overlap between studies from effect sizes resulting from the same sample (López-López et al., 2018).

Last, while we were able to theorize about institutional logics and how they contribute to contextual differences in care settings, more direct quantitative measurement of these phenomena was not evident in the literature and therefore was not able to be analyzed. It is important to note that the analysis was dependent on correlational data, and while associations between care quality, CWBs, and job demands/resource and job strain were found, this does not infer causation.

### Future Directions

Our findings provide tentative support for our theorizing that broader logics may be affecting work design and resource allocation in residential aged care organizations. These perceptions may be affected by market and professional logics within a neoliberal society (Mumby, 2019). However, links between market and professional logics and work design need to be empirically established. Specific research that investigates the impact of market and professional logics on beliefs regarding care worker value and work designer decisions is crucial to advancing a research agenda to better understand resource investment in frontline staff. Future research should explore the potential barriers for organizations to invest in good work design through qualitative studies asking questions such as: what are the roles of institutional logics (such as the market, or professional logics) in creating work design outcomes in residential aged care?

## Conclusion

This study synthesized and quantified the current body of knowledge about whether poor QoC and CWBs were predicted by high job demands, low job resources, and job strain in care settings. We found that lower QoC was predicted by higher job demands, lower job resources, and job strain; and higher CWBs were predicted by high job demands and job strain. In addition, we found a stronger negative relationship between QoC and low job resources in residential aged care versus hospital settings. We argue that these field-level differences are attributable to work design in the residential aged care which is driven by market and professional logics. Further research is required to investigate the impact of institutional logics on work designer decisions, and how this might be facilitating underresourced frontline roles in residential aged care settings.

## Supplementary Material

gnac039_suppl_Supplementary_Material
