# Supplementary material for: Does Context Count? The Association Between Quality of Care and Job Characteristics in Residential Aged Care and Hospital Settings: A Systematic Review and Meta-Analysis
Source: Gerontologist. 2022 Mar 22;63(6):1012–27. doi: 10.1093/geront/gnac039 (PMC10353044; doi:10.1093/geront/gnac039)
Supplement: gnac039_suppl_Supplementary_Material [file gnac039_suppl_supplementary_material.docx]

**Supplementary Table 1.** *Search Terms for Meta-Analysis Using Databases Web of Science, CINAHL, SCOPUS, ABI/INFORM/ProQuest, and Medline*

| Variable | Search terms |
| --- | --- |
| Job strain | AB (Burnout OR burn-out OR "burn out" OR "Occupational stress" OR "workplace stress" OR "work place stress" OR "work-place stress" OR "job stress" OR "work stress" and "work strain" OR "compassion fatigue" OR Depersonalisation OR depersonalization OR "Job strain" OR "emotional exhaustion" OR "mental stress" OR "psychological stress" OR "employee stress" OR "stress disorder*" OR "mental strain" OR "psychological strain" OR "employee strain" OR "Personal accomplishment" OR "workplace strain" OR "work place strain" OR "work-place strain" OR "job strain") |
| Job Stressors and Job Resources | AB ( "Job stressor*" OR "Work stressor*" OR "job demand*" OR "job demands-resource*" OR JDR OR workload or "work demand*" or "work pressure" or "job pressure" or "monotonous work" or "emotional demand*" or "emotional labour" OR "Job enrichment" OR Autonomy OR "decision latitude" OR "job control" OR "work control" OR "decision authority" OR "supervis* support*" OR "social support*" OR "coworker support*" OR "emotional support*" OR "peer support*" OR "job resourc*" OR "role conflict" OR "role ambiguity" OR "role clarity" OR Job insecurity" OR "insecure work*" OR "casual work*" OR casualisation OR "precarious work*" OR "Job uncertainty" OR "uncertain work" OR "time pressure*" OR "work* hours" OR overtime OR "role overload" OR "heavy workload" OR "high demands" OR "shift work*" OR "work schedule" OR "Job overload" OR "work overload" OR "work pressure" OR "trouble* NEAR/3 client" OR "aggress* NEAR/3 client" OR "abus* NEAR/3 client" OR "demand* NEAR/3 client" OR "difficult NEAR/3 client" OR "violent NEAR/3 client" OR "troublesome NEAR/3 resident*" OR "aggress* NEAR/3 resident*" OR "abus* NEAR/3 resident*" OR "demand* NEAR/3 resident*" OR "difficult NEAR/3 resident*" OR "violent NEAR/3 resident*" OR "Skill discretion" OR "effort-reward* imbalance" OR ERI OR "bullying" OR "interpersonal communication" OR "interpersonal relationship*" OR "interpersonal conflict" OR "supervis* conflict" OR "hazard* OR "physical demand*" OR Psychosocial OR "work resource*" OR "work pace" OR "work conflict" OR "task conflict" OR "relationship conflict" OR "procedural justice" OR "organi* justice" OR "procedural injustice" OR "organi* injustice" OR reward OR praise OR recognition) |
| Quality of Care | AB ( "Social car*" OR "respect* NEAR/3 car*" OR "Futile car*" OR "Compass* NEAR/3 Car*" OR "Car* NEAR/3 behavi*" OR "Caring behaviours inventory" OR "caring behaviors inventory" OR "person centered car*" or "person centred car*" or "patient centered car*" or "patient centred car*" or "person focused car*" OR "interpersonal car*" OR "interpersonal relat*") |
| CWBs | AB ("Counter productive work behav*" OR "counterproductive work behav*" OR counterproductivity OR "elder harm*" OR "work hostility" OR "work coercion" OR noncompliance OR abuse OR neglect OR maltreatment OR mistreatment OR misconduct OR misbehaviour OR "abandon* elder*" or "abandon* aged" OR "devian behav*" OR deviance OR "work* deviance" OR "employ* deviance" OR "interpersonal deviance" OR "organisation* deviance" OR "work absen*" OR "work attend*" OR "job absen*" OR "job attend*" OR "work* aggression" OR "work* violence" OR "work* bully*" OR "work threats" OR "work humiliation" OR "work abandonment" OR "work deprivation" OR "work* aggression" OR "organisational aggression" OR "work* harassment" OR "unethical behav*" OR wrongdoing OR wrong-doing OR "productive deviance" OR "slow work" OR sloppy OR "poor quality work" OR "misuse NEAR/3 time" OR "misuse NEAR/3 resourc*" OR "exploit* NEAR/3 elder*" OR "exploit* NEAR/3 aged" OR "assault NEAR/3 elder*" OR "assault NEAR/3 aged" OR "punish* NEAR/3 elder*" OR "punish* NEAR/3 aged" OR "overadministration of drug*" OR "chemical abuse" OR "medic* misconduct" OR "unsafe behavi*r*" OR "unsafe practice" OR "psychological abuse" OR "psychological torment") |
| Setting | AB ( "aged care" or "nursing home" or "residential NEAR/3 aged NEAR/3 care" or "long term care" OR "hospital" OR "geriatric care" ) |
| Population of interest | AB ("worker" OR "staff" OR "personnel" OR "nurse" OR "employee" OR "carer") |
| NOT | NOT "qualitative research" or "qualitative study" "systematic review" or "sexual abuse" or "sexual harrassment" or suicide or "drug trial*" or "family car*" or "informal car*" or "drug treatment" or "epidemiology" or case study" or "law journal" or social welfare" or "social work" or "oral health" or "focus group" or "personality" or "family violence" or "sex differences" or "gender differences" or "infection control" or "descriptive research" or "literature review" or "stress management" or "drug therapy" or "child" or "young adult" or "adolescent" or "teenager*" |
| Search combination | |
| Search combination 1 | Job stressors/Job resources AND Quality of care AND Setting AND Population of interest NOT *list* |
| Search combination 2 | Job stressors/Job resources AND CWBs AND Setting AND Population of interest NOT *list* |
| Search combination 3 | Job strain AND Quality of care AND Setting AND Population of interest NOT *list* |
| Search combination 4 | Job strain AND CWBs AND Setting AND Population of interest NOT *list* |
| Final Export | **TOTAL "combined with OR"** |

STROBE Statement—Checklist of items that should be included in reports of cross-sectional studies

|  | Item No | **Recommendation** |
| --- | --- | --- |
| **Title and abstract** | 1 | (*a*) Indicate the study’s design with a commonly used term in the title or the abstract |
|  |  | (*b*) Provide in the abstract an informative and balanced summary of what was done and what was found |
| Introduction | | |
| Background/rationale | 2 | Explain the scientific background and rationale for the investigation being reported |
| Objectives | 3 | State specific objectives, including any prespecified hypotheses |
| Methods | | |
| Study design | 4 | Present key elements of study design early in the paper |
| Setting | 5 | Describe the setting, locations, and relevant dates, including periods of recruitment, exposure, follow-up, and data collection |
| Participants | 6 | (*a*) Give the eligibility criteria, and the sources and methods of selection of participants |
| Variables | 7 | Clearly define all outcomes, exposures, predictors, potential confounders, and effect modifiers. Give diagnostic criteria, if applicable |
| Data sources/ measurement | 8 | For each variable of interest, give sources of data and details of methods of assessment (measurement). Describe comparability of assessment methods if there is more than one group |
| Bias | 9 | Describe any efforts to address potential sources of bias |
| Study size | 10 | Explain how the study size was arrived at |
| Quantitative variables | 11 | Explain how quantitative variables were handled in the analyses. If applicable, describe which groupings were chosen and why |
| Statistical methods | 12 | (*a*) Describe all statistical methods, including those used to control for confounding |
|  |  | (*b*) Describe any methods used to examine subgroups and interactions |
|  |  | (*c*) Explain how missing data were addressed |
|  |  | (*d*) If applicable, describe analytical methods taking account of sampling strategy |
|  |  | (*e*) Describe any sensitivity analyses |
| Results | | |
| Participants | 13 | (a) Report numbers of individuals at each stage of study—eg numbers potentially eligible, examined for eligibility, confirmed eligible, included in the study, completing follow-up, and analysed |
|  |  | (b) Give reasons for non-participation at each stage |
|  |  | (c) Consider use of a flow diagram |
| Descriptive data | 14 | (a) Give characteristics of study participants (eg demographic, clinical, social) and information on exposures and potential confounders |
|  |  | (b) Indicate number of participants with missing data for each variable of interest |
| Outcome data | 15 | Report numbers of outcome events or summary measures |
| Main results | 16 | (*a*) Give unadjusted estimates and, if applicable, confounder-adjusted estimates and their precision (e.g., 95% confidence interval). Make clear which confounders were adjusted for and why they were included |
|  |  | (*b*) Report category boundaries when continuous variables were categorized |
|  |  | (*c*) If relevant, consider translating estimates of relative risk into absolute risk for a meaningful time period |
| Other analyses | 17 | Report other analyses done—e.g. analyses of subgroups and interactions, and sensitivity analyses |
| Discussion | | |
| Key results | 18 | Summarise key results with reference to study objectives |
| Limitations | 19 | Discuss limitations of the study, taking into account sources of potential bias or imprecision. Discuss both direction and magnitude of any potential bias |
| Interpretation | 20 | Give a cautious overall interpretation of results considering objectives, limitations, multiplicity of analyses, results from similar studies, and other relevant evidence |
| Generalisability | 21 | Discuss the generalisability (external validity) of the study results |
| Other information | | |
| Funding | 22 | Give the source of funding and the role of the funders for the present study and, if applicable, for the original study on which the present article is based |

**Supplementary** **Table 2.** *Quality Assessment using STROBE (Strengthening the Reporting of Observational Studies in Epidemiology)*

| **Study** | **1** | **2** | **3** | **4** | **5** | **6** | **7** | **8** | **9** | **10** | **11** | **12** | **13** | **14** | **15** | **16** | **17** | **18** | **19** | **20** | **21** | **22** | **Quality score** |
| --- | --- | --- | --- | --- | --- | --- | --- | --- | --- | --- | --- | --- | --- | --- | --- | --- | --- | --- | --- | --- | --- | --- | --- |
| Abdelhadi & Drach‐Zahavy (2012) | + | + | + | + | + | + | + | + | + | + | + | + | - | + | + | - | + | + | + | + | + | + | 20/22 |
| Abekah-Nkrumah & Nkrumah (2021) | + | + | + | + | + | + | + | + | - | + | + | - | + | + | + | + | - | + | + | + | - | + | 18/22 |
| Alhalal et al., (2020) | + | + | + | + | + | + | + | + | - | + | + | - | - | + | + | + | - | + | + | + | - | + | 17/22 |
| Alexiou et al., (2021) | + | + | + | + | + | + | + | + | - | - | + | - | - | + | + | + | + | + | + | + | - | + | 17/22 |
| Andela et al., (2018a) | + | + | + | + | + | + | + | + | + | - | + | - | - | + | + | + | + | + | + | + | - | + | 18/22 |
| Bachnick et al., (2018) | + | + | + | + | + | + | + | + | + | - | + | - | + | + | + | + | + | + | + | + | + | + | 20/22 |
| Backman (2016) | + | + | + | + | + | + | + | + | - | - | + | + | - | - | + | - | + | + | + | + | + | + | 17/22 |
| Basar & Basim (2016) | + | + | + | + | + | + | + | + | - | + | + | + | - | + | + | + | + | + | + | + | + | + | 20/22 |
| Bégat et al., (2004) | + | + | - | + | + | + | + | + | - | - | + | - | + | + | - | - | + | + | + | + | - | + | 16/22 |
| Burtson & Stichler, (2010) | + | + | + | + | + | + | + | + | + | + | + | + | + | + | + | + | + | + | + | + | + | + | 22/22 |
| Caspar & O’Rourke  (2008) | + | + | + | + | + | + | + | + | - | + | + | + | + | + | + | + | + | + | + | + | + | - | 20/22 |
| Caspar et al., (2017) | + | + | + | + | + | + | + | + | + | + | + | + | + | + | + | + | + | + | + | + | + | + | 22/22 |
| Chana et al., (2015) | + | + | + | - | + | + | + | - | - | + | + | + | + | + | - | - | - | + | + | + | + | + | 16/22 |
| Chao et al., (2016) | + | + | + | + | + | + | + | + | - | + | + | + | + | + | + | - | + | + | + | + | + | + | 20/22 |
| El-Hneiti (2019) | + | + | + | + | + | + | + | + | - | + | + | + | + | + | + | - | - | + | + | + | + | + | 19/22 |
| Fagbenro (2019) | + | + | + | + | + | + | + | + | - | - | + | - | + | + | + | - | + | + | + | + | + | + | 18/22 |
| Fopma-Loy (1991) | + | + | + | + | + | + | + | + | - | - | + | + | + | + | + | + | + | + | + | + | + | n/a | 20/22 |
| Gountas et al., (2014) | + | + | + | + | + | + | + | + | - | - | + | + | + | + | + | + | + | + | + | + | - | + | 19/22 |
| Harris & Artis (2005) | + | + | + | + | + | + | + | + | - | + | + | + | - | + | + | + | + | + | + | + | + | - | 19/22 |
| Jamal (1984) | + | + | + | + | + | + | + | + | - | - | + | + | + | - | - | + | - | + | + | - | + | - | 15/22 |
| Jarrar et al., (2019) | + | + | - | + | + | + | + | + | - | - | - | - | - | - | + | + | + | + | + | - | - | - | 12/22 |
| Kaur et al., (2013) | + | + | + | + | + | + | + | + | - | - | + | + | + | + | + | - | + | + | + | + | + | + | 19/22 |
| Kazemi & Corlin (2021) | + | + | + | + | + | + | + | + | - | + | + | - | - | + | + | + | + | + | - | + | + | - | 17/22 |
| Kim & Park (2019) | n/a* | n/a* | + | + | + | + | + | + | - | + | + | + | + | + | + | + | + | + | + | - | - | - | 18(?)/22 |
| Lee & Pak (2016) | + | + | n/a* | n/a* | n/a* | n/a* | n/a* | n/a* | n/a* | n/a* | n/a* | n/a* | + | + | + | + | + | n/a* | n/a* | n/a* | n/a* | n/a* | 7(?)/22 |
| Lee (2004) | + | + | + | + | + | + | + | + | - | + | + | + | + | + | + | + | + | + | + | + | + | n/a | 21/22 |
| Liu & Aungsuroch (2017) | + | + | + | + | + | + | + | + | - | + | + | + | + | + | + | - | + | + | + | + | - | - | 18/22 |
| McLinton (2019) | + | + | + | + | + | + | + | + | + | + | + | + | + | - | + | + | + | + | + | + | + | + | 21/22 |
| McNeil et al., (2019) | + | + | + | + | + | + | + | + | + | + | + | + | + | + | + | - | + | + | + | + | - | + | 20/22 |
| Motowidlo et al., (1986) | + | + | + | + | - | + | + | + | - | - | + | + | - | - | + | - | + | + | + | + | - | - | 15/22 |
| Neuberg et al., (2017) | + | + | + | + | + | + | + | + | - | - | + | - | - | + | + | + | - | + | + | + | - | - | 15/22 |
| Panunto et al., (2013) | + | + | + | + | + | + | + | + | - | - | + | + | + | + | + | - | + | + | + | + | - | - | 17/22 |
| Rathert et al., (2020) | + | + | + | + | + | + | + | + | - | - | + | + | - | - | + | + | + | + | + | + | - | - | 16/22 |
| Sarafis et al., (2016) | + | + | + | + | + | + | + | + | - | - | + | + | + | + | + | + | + | + | + | + | + | + | 20/22 |
| Sarwar et al., (2020) | + | + | + | + | + | + | + | + | + | + | + | + | + | + | + | + | + | + | + | + | - | + | 21/22 |
| Shafique et al., (2021) | + | + | + | + | + | + | + | + | + | + | + | + | + | + | + | + | + | + | + | + | + | - | 21/22 |
| Skoldunger et al., (2020) | + | + | + | + | + | + | + | + | - | - | + | + | + | + | + | + | + | + | + | + | + | + | 20/22 |
| Sullivan et al., (2019) | + | + | + | + | + | + | + | + | - | - | + | + | + | + | + | - | + | + | + | + | + | + | 20/22 |
| Van Bogaert et al., (2009) | + | + | + | + | + | + | + | + | - | + | + | + | + | + | + | - | + | + | + | + | - | + | 19/22 |
| Van Bogaert et al., (2013) | + | + | + | + | + | + | + | + | - | - | + | + | - | + | + | + | + | + | + | + | + | + | 19/22 |
| Van Diepen et al.,  (2021) | + | + | + | + | + | + | + | + | - | + | + | + | + | + | + | + | + | + | + | + | - | + | 20/22 |
| Zuniga et al.,  (2015) | + | + | + | + | + | + | + | + | - | - | + | + | + | + | + | + | + | + | + | + | - | + | 19/22 |

*not in English

Quality scoring sheet based on quartiles.

| Score | Classification |
| --- | --- |
| 17-22 | High |
| 11- 17 | Moderate |
| 6 - 11 | Low |
| < 5 | Unacceptable |

Supplementary Figure 1: Funnel plots for correlations

| Funnel plot for studies measuring the association between QOC and job demands | Funnel plot for studies measuring the association between CWBs and job demands. |
| --- | --- |
| 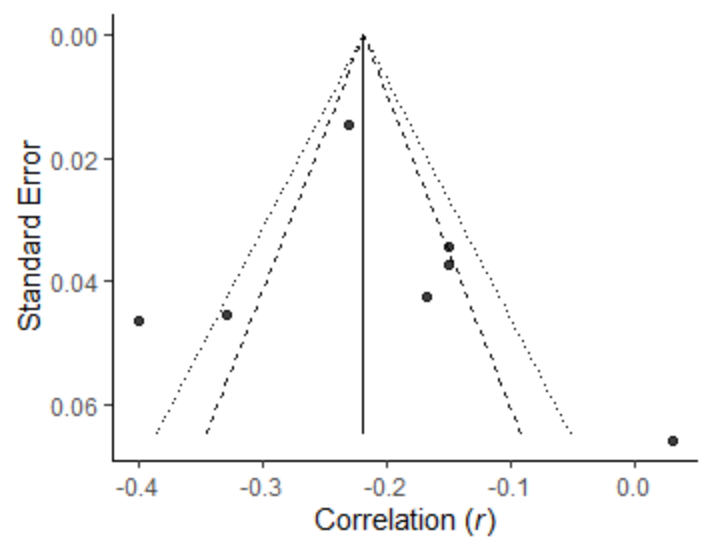 | 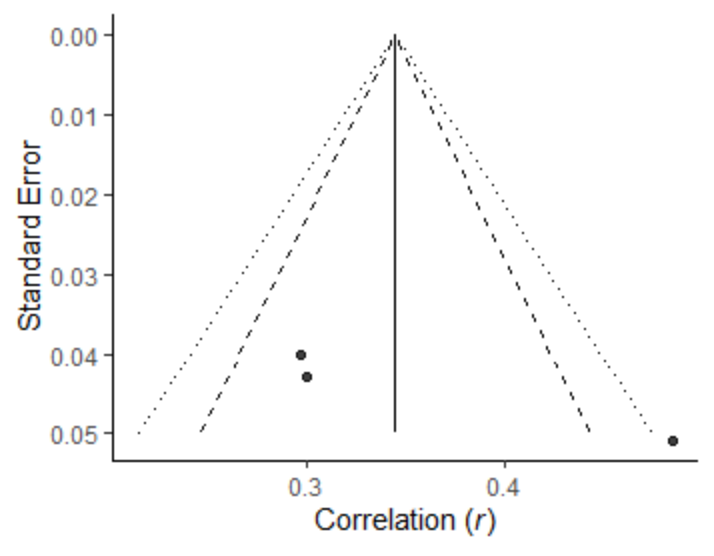 |
| Funnel plot for studies measuring the association between QOC and low job resources. |  |
| 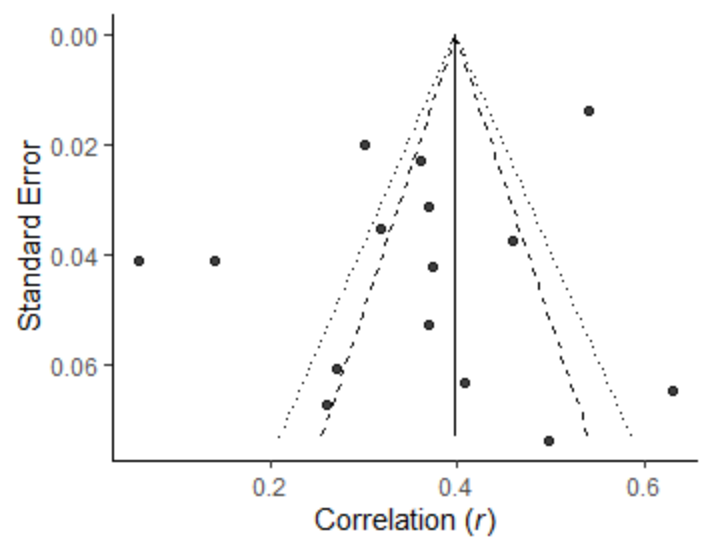 |  |
| Funnel plot for studies measuring the association between QOC and job strain. | Funnel plot for studies measuring the association between CWBs and job strain. |
| 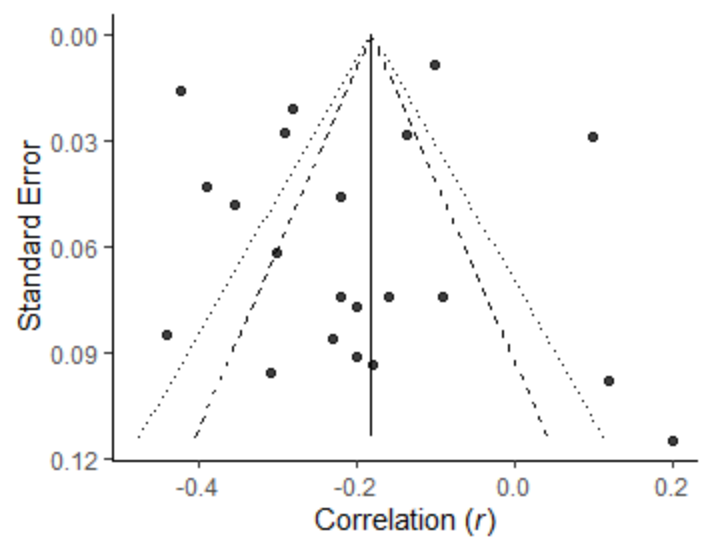 | 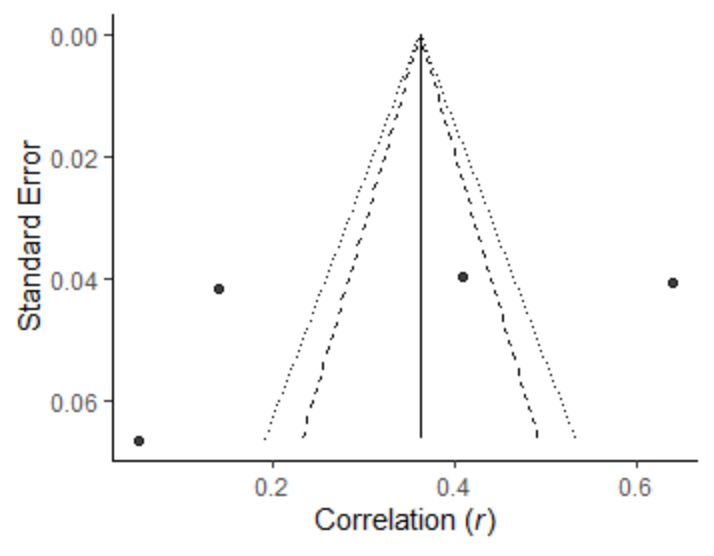 |

**Supplementary** **Figure 2.** Forest Plots for Correlations

| Forest plot for corrected correlations for studies measuring the association between QOC and job demands. | Forest plot for corrected correlations for studies measuring the association between CWBs and job demands. |
| --- | --- |
| 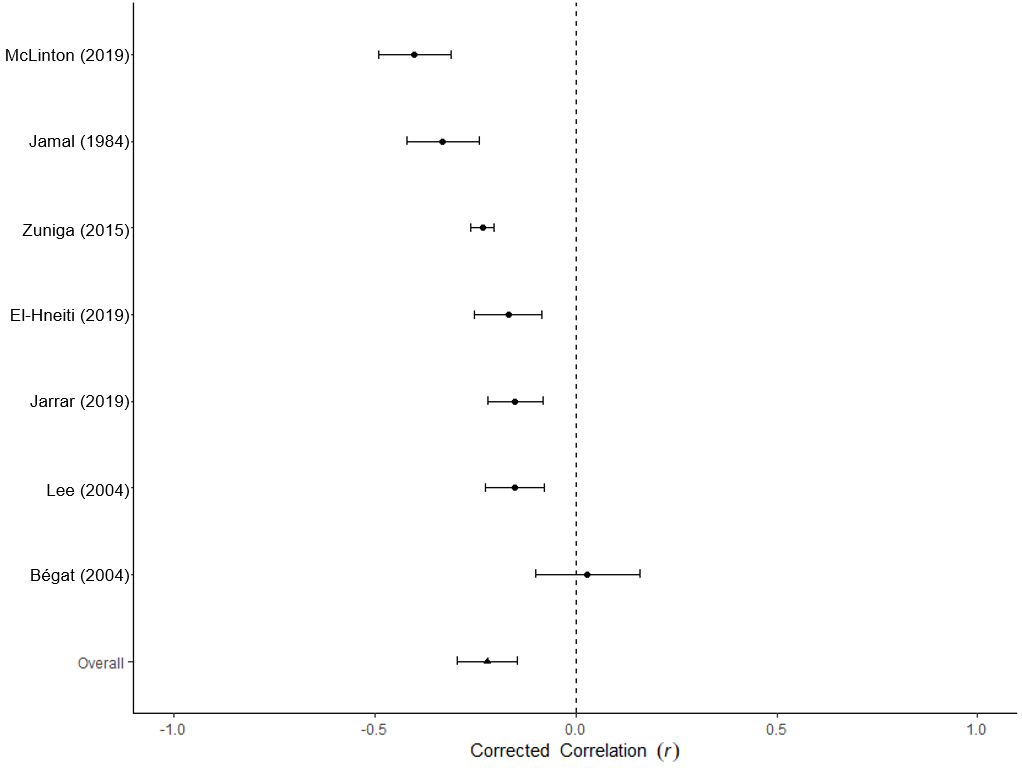 | 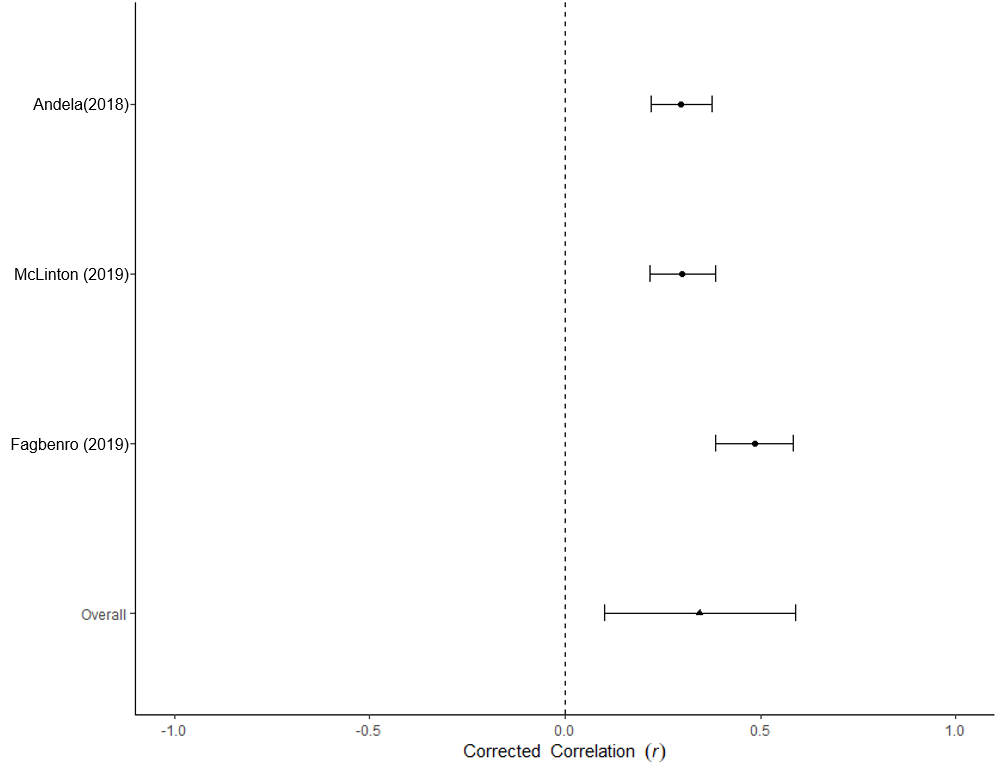 |
| Forest plot for corrected correlations for studies measuring the association between QOC and low job resources. |  |
| 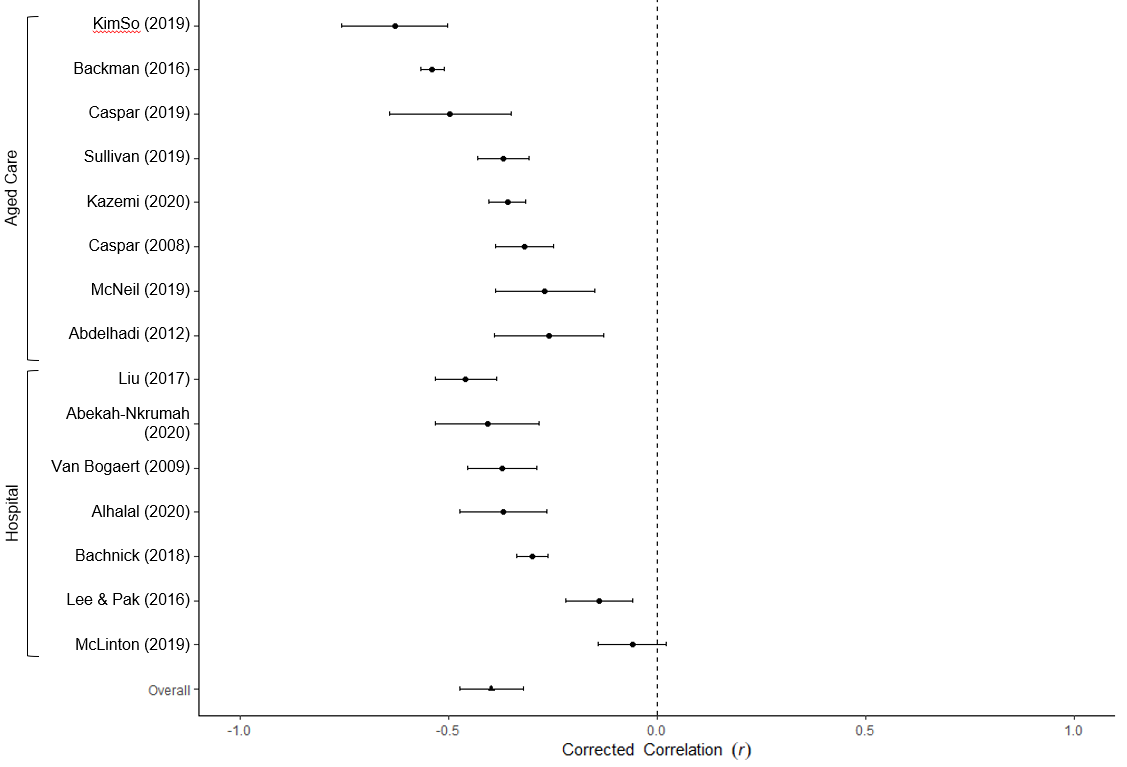 |  |
| Forest plot for corrected correlations for studies measuring the association between QOC and job strain. | Forest plot for corrected correlations for studies measuring the association between CWBs and job strain. |
| 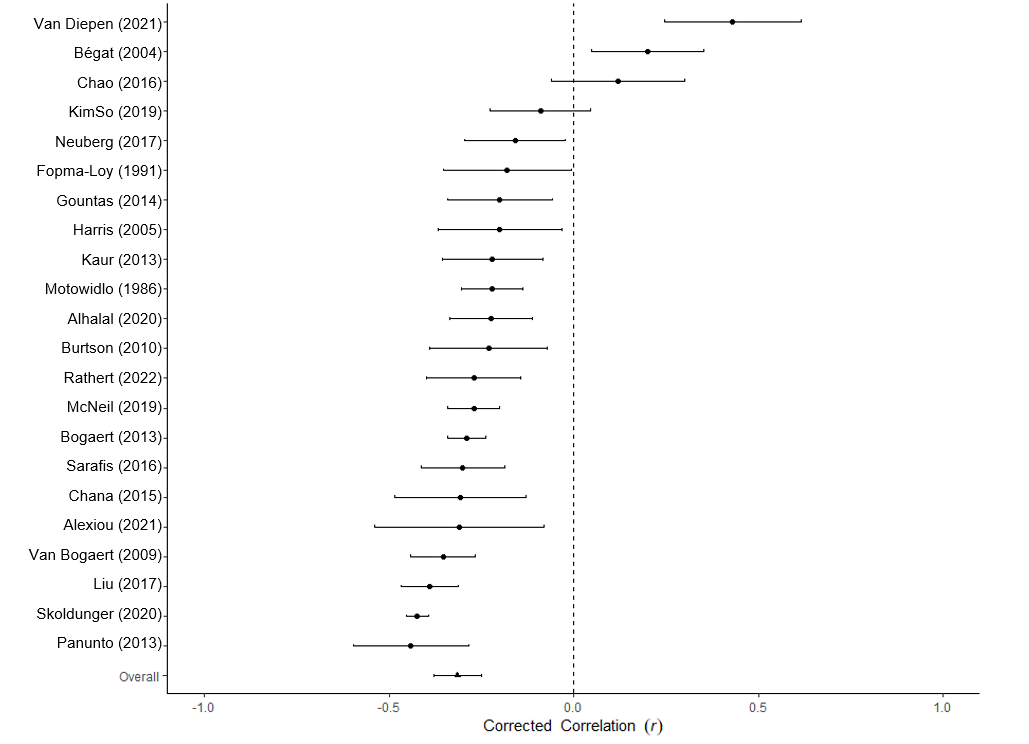 | 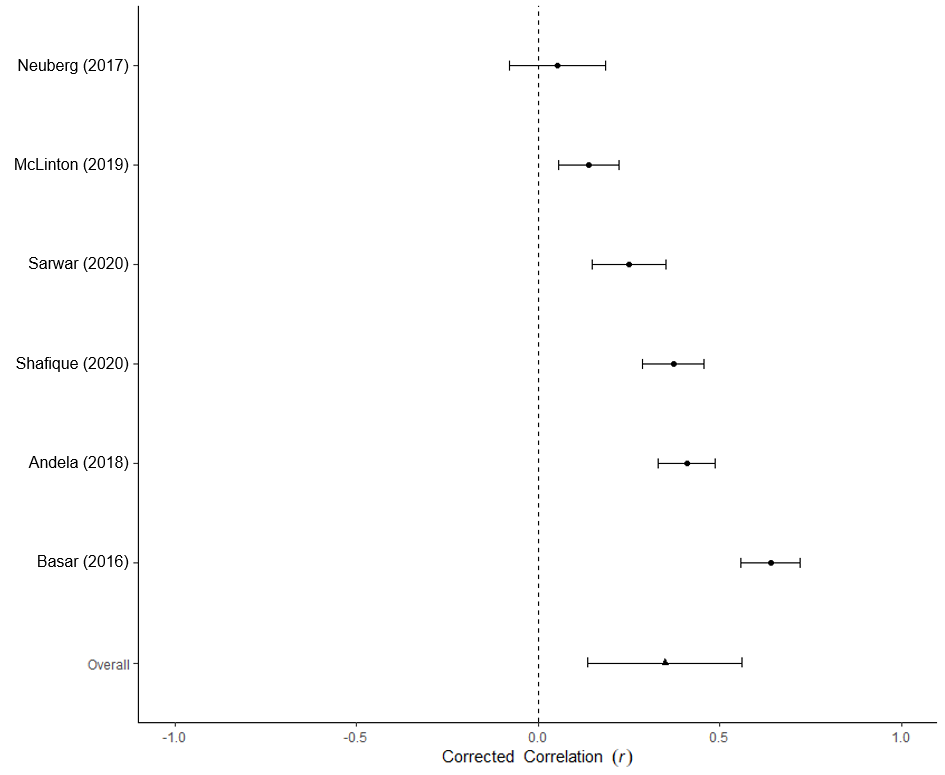 |

**Tests of residual heterogeneity**

We conducted a moderation analysis for the study-level variable of specific measures of job strain in order to account for unexplained heterogeneity. Job strain was categorized as either burnout (operationalized using a burnout measure such as the Maslach burnout inventory or the ProQol), or a generic job strain measure operationalized as job strain or job stress. The specific measures are highlighted in Table 1. Previous studies have justified treating burnout, job strain and job stress as a unitary variable, but it is still possible that there may be differences between the constructs and the way they are measured. While some of the residual heterogeneity was accounted for with a decreased *I^2^* of 76.46%, the moderation was not significant, F(2, 19) = 4.05, *p*=0.13, *k*=22, indicating differences in job strain measurement did not moderate main effects.

**List of included studies**

Abdelhadi, N., & Drach-Zahavy, A. (2012). Promoting patient care: work engagement as a mediator between ward service climate and patient-centred care*. Journal of Advanced Nursing*, 68(6), 1276–1287. doi:10.1111/j.1365-2648.2011.05834.x

Abekah-Nkrumah, G., & Nkrumah, J. (2021). Perceived work environment and patient-centered behavior: A study of selected district hospitals in the central region of Ghana. *PLOS ONE*, 16(1), e0244726. doi:10.1371/journal.pone.0244726

Alexiou, E., Kjellberg, I. L., & Wijk, H. (2021). Sustainable implementation of person-centred care in residential care facilities: hindering and supporting factors when improving incontinence care. *Nursing and Residential Care*, 23(1), 1–14. doi:10.12968/nrec.2021.23.1.5

Alhalal, E., Alrashidi, L. M., & Alanazi, A. N. (2020). Predictors of patient‐centered care provision among nurses in acute care setting. *Journal of Nursing Management.* doi:10.1111/jonm.13100

Andela, M., Truchot, D., & Huguenotte, V. (2018). Work Environment and Elderly Abuse in Nursing Homes: The Mediating Role of Burnout*. Journal of Interpersonal Violence,* 36(11-12), 5709–5729. doi:10.1177/0886260518803606

Bachnick, S., Ausserhofer, D., Baernholdt, M., & Simon, M. (2018). Patient-centered care, nurse work environment and implicit rationing of nursing care in Swiss acute care hospitals: A cross-sectional multi-center study. *International Journal of Nursing Studies*, 81, 98–106. doi:10.1016/j.ijnurstu.2017.11.007

Backman, A., Sjögren, K., Lindkvist, M., Lövheim, H., & Edvardsson, D. (2016). Towards person-centredness in aged care - exploring the impact of leadership. *Journal of Nursing Management*, 24(6), 766–774. doi:10.1111/jonm.12380

Basar, U., & Basim, N. (2016). A cross‐sectional survey on consequences of nurses' burnout: moderating role of organizational politics. *Journal of Advanced Nursing*, *72*(8), 1838-1850. doi:10.1111/jan.12958

Begat, I., Ikeda, N., Amemiya, T., Emiko, K., Iwasaki, A., & Severinsson, E. (2004). Comparative study of perceptions of work environment and moral sensitivity among Japanese and Norwegian nurses. *Nursing and Health Sciences*, 6(3), 193–200. doi:10.1111/j.1442-2018.2004.00192.x

Burtson, P. L., & Stichler, J. F. (2010). Nursing work environment and nurse caring: relationship among motivational factors. *Journal of Advanced Nursing*, 66(8), 1819–1831. doi:10.1111/j.1365-2648.2010.05336.x

Caspar, S., Le, A., & McGilton, K. S. (2017). The Influence of Supportive Supervisory Practices and Health Care Aides’ Self-Determination on the Provision of Person-Centered Care in Long-Term Care Facilities. *Journal of Applied Gerontology*, 38(11), 1564–1582. doi:10.1177/0733464817750275

Caspar, S., & O’Rourke, N. (2008). The Influence of Care Provider Access to Structural Empowerment on Individualized Care in Long-Term-Care Facilities. *The Journals of Gerontology Series B: Psychological Sciences and Social Sciences,* 63(4), S255–S265. doi:10.1093/geronb/63.4.s255

Chana, N., Kennedy, P., & Chessell, Z. J. (2015). Nursing staffs' emotional well‐being and caring behaviours. *Journal of Clinical Nursing*, *24*(19-20), 2835-2848. doi: 10.1111/jocn.12891

Chao, M., Shih, C. T., & Hsu, S. F. (2016). Nurse occupational burnout and patient‐rated quality of care: The boundary conditions of emotional intelligence and demographic profiles. *Japan Journal of Nursing Science*, 13(1), 156-165. doi: 10.1111/jjns.12100

El‐Hneiti, M., Shaheen, A. M., Bani Salameh, A., Al-dweeri Rami Mohammad, Al‐Hussami, M., Alfaouri, F. T., & Ahmad, M. (2019). An explorative study of workplace violence against nurses who care for older people. *Nursing Open*, 7(1), 285–293. doi:10.1002/nop2.389

Fagbenro, D. A. (2019). Role Ambiguity and Organizational Justice as the Predictors of Unethical Behavior Among Nurses. *Journal of Client-Centered Nursing Care*, 81–86. doi:10.32598/jccnc.5.2.81

Fopma-Loy, J. (1991). Predictors of caregiving behaviors of formal caregivers of institutionalized people with dementing illnesses. *Unpublished doctoral dissertation, Indiana University School of Nursing, Indianapolis*.

Gountas, S., Gountas, J., Soutar, G., & Mavondo, F. (2014). Delivering good service: personal resources, job satisfaction and nurses' 'customer' (patient) orientation. *Journal of advanced nursing*, 70(7), 1553–1563. <https://doi.org/10.1111/jan.12308>

Harris, E. G., & Artis, A. B. (2005). Exploring Patient, Co-Worker, and Management Burnout in Health Care. *Health Marketing Quarterly*, 22(3), 3–20. doi:10.1300/j026v22n03_02

Jamal, M. (1984). Job stress and job performance controversy: An empirical assessment. *Organizational Behavior and Human Performance,* 33(1), 1–21. doi:10.1016/0030-5073(84)90009-6

Jarrar, M., Minai, M. S., Al-Bsheish, M., Meri, A., & Jaber, M. (2019). Hospital nurse shift length, patient-centered care, and the perceived quality and patient safety. *The International Journal of Health Planning and Management*, 34(1), 387-396. doi:10.1002/hpm.2656

Kaur, D., Sambasivan, M., & Kumar, N. (2013). Effect of spiritual intelligence, emotional intelligence, psychological ownership and burnout on caring behaviour of nurses: a cross-sectional study. *Journal of Clinical Nursing*, 22(21-22), 3192–3202. doi:10.1111/jocn.12386

Kazemi, A., & Elfstrand Corlin, T. (2021). Linking supportive leadership to satisfaction with care: proposing and testing a service-profit chain inspired model in the context of elderly care. *Journal of Health Organization and Management,* 35(4), 492–510. doi:10.1108/jhom-10-2020-0393

Kim, S. B., & Park, Y. (2019). Factors associated with Person-centered Care for Elderly in Long-term Care Hospital Nurses. *Korean Journal of Adult Nursing*, 31(6), 618. doi:10.7475/kjan.2019.31.6.618

Lee, J. Y., & Pak, S. Y. (2016). The impacts of nurses' psycho-social health and social support from colleagues on patient caring ability. *Journal of Korean Academy of Nursing Administration*, *22*(5), 461-470. doi: 10.11111/jkana.2016.22.5.461

Lee, M.-H. (2004). *The impact of work stressors on caring behaviors and professional job satisfaction among nurses in Taiwan*, University of California, San Francisco.

Liu, Y., & Aungsuroch, Y. (2017). Factors influencing nurse-assessed quality nursing care: A cross-sectional study in hospitals. *Journal of Advanced Nursing,* 74(4), 935–945. doi:10.1111/jan.13507

McLinton, S. S., Afsharian, A., Dollard, M. F., & Tuckey, M. R. (2019). The dynamic interplay of physical and psychosocial safety climates in frontline healthcare. *Stress and Health*, 35(5), 650–664. doi:10.1002/smi.2898

McNeil, N., Bartram, T., Cregan, C., Ellis, J., & Cooke, F. L. (2019). Caring for aged people: The influence of personal resilience and workplace climate on “doing good” and “feeling good.” *Journal of Advanced Nursing,* 75(7), 1450–1461. doi:10.1111/jan.13935

Motowidlo, S. J., Packard, J. S., & Manning, M. R. (1986). Occupational stress: Its causes and consequences for job performance. *Journal of Applied Psychology*, 71(4), 618–629. doi:10.1037/0021-9010.71.4.618

Neuberg, M., Železnik, D., Meštrović, T., Ribić, R., & Kozina, G. (2017). Is the burnout syndrome associated with elder mistreatment in nursing homes: results of a cross-sectional study among nurses. *Archives of Industrial Hygiene and Toxicology*, 68(3), 190–197. doi:10.1515/aiht-2017-68-2982

Panunto, M. R., & Guirardello, E. de B. (2013). Professional nursing practice: environment and emotional exhaustion among intensive care nurses. *Revista Latino-Americana de Enfermagem*, 21(3), 765–772. doi:10.1590/s0104-11692013000300016

Rathert, C., Ishqaidef, G., & Porter, T. H. (2020). Caring work environments and clinician emotional exhaustion. *Health Care Management Review*, 47(1), 58–65. doi:10.1097/hmr.0000000000000294

Sarafis, P., Rousaki, E., Tsounis, A., Malliarou, M., Lahana, L., Bamidis, P., … Papastavrou, E. (2016). The impact of occupational stress on nurses’ caring behaviors and their health related quality of life. *BMC Nursing*, 15(1). doi:10.1186/s12912-016-0178-y

Sarwar, A., Khan, J., Muhammad, L., Mubarak, N., & Jaafar, M. (2021). Relationship between organisational dehumanization and nurses' deviant behaviours: A moderated mediation model. *Journal of Nursing Management*, *29*(5), 1036-1045. doi: 10.1111/jonm.13241

Shafique, I., Qammar, A., Kalyar, M. N., Ahmad, B., & Mushtaq, A. (2020). Workplace ostracism and deviant behaviour among nurses: a parallel mediation model. *Journal of Asia Business Studies*, 15(1), 50–71. doi:10.1108/jabs-03-2020-0096

Sköldunger, A., Sandman, P.-O., & Backman, A. (2020). Exploring person-centred care in relation to resource utilization, resident quality of life and staff job strain – findings from the SWENIS study. *BMC Geriatrics,* 20(1). doi:10.1186/s12877-020-01855-7

Sullivan, J. L., Weinburg, D. B., Gidmark, S., Engle, R. L., Parker, V. A., & Tyler, D. A. (2019). Collaborative capacity and patient-centered care in the Veterans’ Health Administration Community Living Centers. *International Journal of Care Coordination*, 22(2), 90–99. doi:10.1177/2053434519858028

Van Bogaert, P., Kowalski, C., Weeks, S. M., Van heusden Danny, & Clarke, S. P. (2013). The relationship between nurse practice environment, nurse work characteristics, burnout and job outcome and quality of nursing care: A cross-sectional survey. *International Journal of Nursing Studies,* 50(12), 1667–1677. doi:10.1016/j.ijnurstu.2013.05.010

Van Bogaert, P., Meulemans, H., Clarke, S., Vermeyen, K., & Van de Heyning, P. (2009). Hospital nurse practice environment, burnout, job outcomes and quality of care: test of a structural equation model. *Journal of Advanced Nursing*, 65(10), 2175–2185. doi:10.1111/j.1365-2648.2009.05082.x

Van Diepen, C., Fors, A., Ekman, I., Bertilsson, M., & Hensing, G. (2021). Associations between person‐centred care and job strain, stress of conscience, and intent to leave among hospital personnel. *Journal of Clinical Nursing*, 31(5-6), 612–622. doi:10.1111/jocn.15919

Zúñiga, F., Ausserhofer, D., Hamers, J. P., Engberg, S., Simon, M., & Schwendimann, R. (2015). Are staffing, work environment, work stressors, and rationing of care related to care Workers' perception of quality of care? A cross-sectional study. *Journal of the American Medical Directors Association*, *16*(10), 860-866. doi: 10.1016/j.jamda.2015.04.012

# **PRISMA Guidelines**

| **Section and Topic** | **Item #** | **Checklist item** | **Location where item is reported** |
| --- | --- | --- | --- |
| **TITLE** | | |  |
| Title | 1 | Identify the report as a systematic review. | Pg 1 |
| **ABSTRACT** | | |  |
| Abstract | 2 | See the PRISMA 2020 for Abstracts checklist. | Pg 2 |
| **INTRODUCTION** | | |  |
| Rationale | 3 | Describe the rationale for the review in the context of existing knowledge. | Pg 4 |
| Objectives | 4 | Provide an explicit statement of the objective(s) or question(s) the review addresses. | Pg 4-5 |
| **METHODS** | | |  |
| Eligibility criteria | 5 | Specify the inclusion and exclusion criteria for the review and how studies were grouped for the syntheses. | Pg 13 |
| Information sources | 6 | Specify all databases, registers, websites, organisations, reference lists and other sources searched or consulted to identify studies. Specify the date when each source was last searched or consulted. | Pg 13 |
| Search strategy | 7 | Present the full search strategies for all databases, registers and websites, including any filters and limits used. | Pg 13 |
| Selection process | 8 | Specify the methods used to decide whether a study met the inclusion criteria of the review, including how many reviewers screened each record and each report retrieved, whether they worked independently, and if applicable, details of automation tools used in the process. | Pg 14-15 |
| Data collection process | 9 | Specify the methods used to collect data from reports, including how many reviewers collected data from each report, whether they worked independently, any processes for obtaining or confirming data from study investigators, and if applicable, details of automation tools used in the process. | Pg 13 |
| Data items | 10a | List and define all outcomes for which data were sought. Specify whether all results that were compatible with each outcome domain in each study were sought (e.g. for all measures, time points, analyses), and if not, the methods used to decide which results to collect. | Pg 14-15 |
|  | 10b | List and define all other variables for which data were sought (e.g. participant and intervention characteristics, funding sources). Describe any assumptions made about any missing or unclear information. | Pg 14-15 |
| Study risk of bias assessment | 11 | Specify the methods used to assess risk of bias in the included studies, including details of the tool(s) used, how many reviewers assessed each study and whether they worked independently, and if applicable, details of automation tools used in the process. | Pg 17 |
| Effect measures | 12 | Specify for each outcome the effect measure(s) (e.g. risk ratio, mean difference) used in the synthesis or presentation of results. | Pg 18 main effects |
| Synthesis methods | 13a | Describe the processes used to decide which studies were eligible for each synthesis (e.g. tabulating the study intervention characteristics and comparing against the planned groups for each synthesis (item #5). | Pg 15-16 |
|  | 13b | Describe any methods required to prepare the data for presentation or synthesis, such as handling of missing summary statistics, or data conversions. | Pg 15-16 |
|  | 13c | Describe any methods used to tabulate or visually display results of individual studies and syntheses. | Pg 15-16 |
|  | 13d | Describe any methods used to synthesize results and provide a rationale for the choice(s). If meta-analysis was performed, describe the model(s), method(s) to identify the presence and extent of statistical heterogeneity, and software package(s) used. | Pg 17, pg 55 |
|  | 13e | Describe any methods used to explore possible causes of heterogeneity among study results (e.g. subgroup analysis, meta-regression). | Pg 17 |
|  | 13f | Describe any sensitivity analyses conducted to assess robustness of the synthesized results. | Pg 17 |
| Reporting bias assessment | 14 | Describe any methods used to assess risk of bias due to missing results in a synthesis (arising from reporting biases). | Pg 17 |
| Certainty assessment | 15 | Describe any methods used to assess certainty (or confidence) in the body of evidence for an outcome. | Pg 18-19 |
| **RESULTS** | | |  |
| Study selection | 16a | Describe the results of the search and selection process, from the number of records identified in the search to the number of studies included in the review, ideally using a flow diagram. | Pg 41 figure 1 |
|  | 16b | Cite studies that might appear to meet the inclusion criteria, but which were excluded, and explain why they were excluded. | Pg 13 |
| Study characteristics | 17 | Cite each included study and present its characteristics. | Pg 36-39 Table 1 |
| Risk of bias in studies | 18 | Present assessments of risk of bias for each included study. | Pg 49-54 supp materials |
| Results of individual studies | 19 | For all outcomes, present, for each study: (a) summary statistics for each group (where appropriate) and (b) an effect estimate and its precision (e.g. confidence/credible interval), ideally using structured tables or plots. | Pg 40-41 Table 2 |
| Results of syntheses | 20a | For each synthesis, briefly summarise the characteristics and risk of bias among contributing studies. | Pg 49-51 supp materials |
|  | 20b | Present results of all statistical syntheses conducted. If meta-analysis was done, present for each the summary estimate and its precision (e.g. confidence/credible interval) and measures of statistical heterogeneity. If comparing groups, describe the direction of the effect. | Pg 40-41 Table 2 |
|  | 20c | Present results of all investigations of possible causes of heterogeneity among study results. | Pg 55 |
|  | 20d | Present results of all sensitivity analyses conducted to assess the robustness of the synthesized results. | Pg 15-16 |
| Reporting biases | 21 | Present assessments of risk of bias due to missing results (arising from reporting biases) for each synthesis assessed. | Pg 49- 50 supplementary materials |
| Certainty of evidence | 22 | Present assessments of certainty (or confidence) in the body of evidence for each outcome assessed. | Pg 40 Table 2 |
| **DISCUSSION** | | |  |
| Discussion | 23a | Provide a general interpretation of the results in the context of other evidence. | Pg 19-26 |
|  | 23b | Discuss any limitations of the evidence included in the review. | Pg 24-25 |
|  | 23c | Discuss any limitations of the review processes used. | Pg 24-25 |
|  | 23d | Discuss implications of the results for practice, policy, and future research. | Pg 22-24 |
| **OTHER INFORMATION** | | |  |
| Registration and protocol | 24a | Provide registration information for the review, including register name and registration number, or state that the review was not registered. | Pg 12 |
|  | 24b | Indicate where the review protocol can be accessed, or state that a protocol was not prepared. | Pg 12 |
|  | 24c | Describe and explain any amendments to information provided at registration or in the protocol. | n/a |
| Support | 25 | Describe sources of financial or non-financial support for the review, and the role of the funders or sponsors in the review. | Pg 3 |
| Competing interests | 26 | Declare any competing interests of review authors. | Pg 3 |
| Availability of data, code and other materials | 27 | Report which of the following are publicly available and where they can be found: template data collection forms; data extracted from included studies; data used for all analyses; analytic code; any other materials used in the review. | 36-39 Table 1 |

*From:*  Page MJ, McKenzie JE, Bossuyt PM, Boutron I, Hoffmann TC, Mulrow CD, et al. The PRISMA 2020 statement: an updated guideline for reporting systematic reviews. BMJ 2021;372:n71. doi: 10.1136/bmj.n71

For more information, visit: <http://www.prisma-statement.org/>
